# Building Emotional Awareness and Mental Health (BEAM): study protocol for a phase III randomized controlled trial of the BEAM app-based program for mothers of children 18–36 months

**DOI:** 10.1186/s13063-022-06512-5

**Published:** 2022-09-05

**Authors:** E. Bailin Xie, Kaeley M. Simpson, Kristin A. Reynolds, Ryan J. Giuliano, Jennifer L. P. Protudjer, Melanie Soderstrom, Shannon Sauer-Zavala, Gerald F. Giesbrecht, Catherine Lebel, Anna L. Mackinnon, Charlie Rioux, Lara Penner-Goeke, Makayla Freeman, Marlee R. Salisbury, Lianne Tomfohr-Madsen, Leslie E. Roos

**Affiliations:** 1 Department of Psychology, University of Calgary, 2500 University Dr. NW, Calgary, AB T2N 1N4 Canada; 2 University of Manitoba, Winnipeg, Canada; 3 Children’s Hospital Research Institute of Manitoba, Winnipeg, Canada; 4 University of Kentucky, Lexington, USA; 5 York University, Toronto, Canada

**Keywords:** Parenting, Maternal, Mental health, Psychoeducation, RCT, Depression

## Abstract

**Background:**

The prevalence of maternal depression and anxiety has increased during the COVID-19 pandemic, and pregnant individuals are experiencing concerningly elevated levels of mental health symptoms worldwide. Many individuals may now be at heightened risk of postpartum mental health disorders. There are significant concerns that a cohort of children may be at-risk for impaired self-regulation and mental illness due to elevated exposure to perinatal mental illness. With both an increased prevalence of depression and limited availability of services due to the pandemic, there is an urgent need for accessible eHealth interventions for mothers of young children. The aims of this trial are to evaluate the efficacy of the Building Emotion Awareness and Mental Health (BEAM) app-based program for reducing maternal depression symptoms (primary outcome) and improve anxiety symptoms, parenting stress, family relationships, and mother and child functioning (secondary outcomes) compared to treatment as usual (TAU).

**Methods:**

A two-arm randomized controlled trial (RCT) with repeated measures will be used to evaluate the efficacy of the BEAM intervention compared to TAU among a sample of 140 mothers with children aged 18 to 36 months, who self-report moderate-to-severe symptoms of depression and/or anxiety. Individuals will be recruited online, and those randomized to the treatment group will participate in 10 weeks of psychoeducation modules, an online social support forum, and weekly group teletherapy sessions. Assessments will occur at 18–36 months postpartum (pre-test, T1), immediately after the last week of the BEAM intervention (post-test, T2), and at 3 months after the intervention (follow-up, T3).

**Discussion:**

eHealth interventions have the potential to address elevated maternal mental health symptoms, parenting stress, and child functioning concerns during and after the COVID-19 pandemic and to provide accessible programming to mothers who are in need of support. This RCT will build on an open pilot trial of the BEAM program and provide further evaluation of this evidence-based intervention. Findings will increase our understanding of depression in mothers with young children and reveal the potential for long-term improvements in maternal and child health and family well-being.

**Trial registration:**

ClinicalTrials.govNCT05306626. Registered on April 1, 2022

## Administrative information

Note: The numbers in curly brackets in this protocol refer to SPIRIT checklist item numbers. The order of the items has been modified to group similar items (see http://www.equator-network.org/reporting-guidelines/spirit-2013-statement-defining-standard-protocol-items-for-clinical-trials/).

**Table:** 

Title {1}	Building Emotional Awareness and Mental Health (BEAM): study protocol for a randomized controlled trial of the BEAM app-based program for mothers of children 18–36 months
Trial registration {2a and 2b}	The trial was registered with ClinicalTrials.gov (NCT05306626) on April 1, 2022. All items from the WHO Trial Registration Dataset can be found within the protocol.
Protocol version {3}	[May, 2022] (Version 1)
Funding {4}	This work was supported by funding from Research Manitoba and the Children’s Hospital Research Institute of Manitoba (July 2020 - July 2022).
Author details {5a}	E. Bailin Xie, University of Calgary Kaeley M. Simpson, University of Manitoba Kristin A. Reynolds, University of Manitoba Ryan J. Giuliano, University of Manitoba Jennifer LP Protudjer, University of Manitoba, Children’s Hospital Research Institute of Manitoba Melanie Soderstrom, University of Manitoba Shannon Sauer-Zavala, University of Kentucky Gerald F. Giesbrecht, University of Calgary Catherine Lebel, University of Calgary Anna L. Mackinnon, University of Calgary Charlie Rioux, University of Manitoba, Children’s Hospital Research Institute of Manitoba, and University of Calgary Lara Penner-Goeke, University of Manitoba Makayla Freeman, University of Calgary Marlee R. Salisbury, York University Leslie Roos, University of Manitoba Lianne Tomfohr-Madsen, University of Calgary **Authors’ contributions** LER, LTM, AM, CR, CL, GG, SSZ, MRS, JP, RJG, and KR contributed to the conceptualization and design of the trial. LER, LTM, AM, CR, EBX, KS, LPG, MF, and MRS contributed to the preparation of the materials and will contribute to conducting the trial. EBX prepared the first draft of the protocol. All authors reviewed and approved the final submission.
Name and contact information for the trial sponsor {5b}	Leslie Roos Department of Psychology, University of Manitoba University of Manitoba, 66 Chancellors Cir, Winnipeg, MB, R3T 2N2. E-mail: leslie.roos@umanitoba.ca. Please contact Dr. Leslie Roos for public and scientific queries.
Role of sponsor {5c}	This funding source had no role in the design of this study and will not have any role during its execution, analyses, interpretation of the data, or decision to submit results.

## Introduction

### Background and rationale {6a}

The COVID-19 pandemic, including the social and public health restrictions implemented to limit the spread of the disease, has resulted in unprecedented stress for families. Physical isolation from support and social networks, economic uncertainty, and additional childcare responsibilities can have unexpected and pernicious effects. Infectious disease outbreaks can increase psychological distress [1, 2] and people in the perinatal period may be especially vulnerable to the negative psychological effects of the COVID-19 pandemic [3, 4]. The COVID-19 pandemic has disproportionally impacted individuals who identify as female, particularly if they are caregivers [5, 6]. Maternal mental health symptoms in the first few years following childbirth are common [7–10] and can have intergenerational impacts on mother and baby. Depression and anxiety, which are the most common perinatal mental health concerns, are risk factors for developing insecure mother-child attachments and higher levels of social, emotional, mental, and behavioral challenges in children [11, 12].

The prevalence of maternal depression and anxiety has increased during the COVID-19 pandemic in Canada and around the world [13–15]. Evidence from the Pregnancy During the COVID-19 Pandemic study has highlighted concerningly elevated levels of mental illness in Canada, with 37% and 57% of pregnant individuals reporting clinically-elevated symptoms of depression and anxiety, respectively [16]. These prevalence rates are much higher than what is normally expected during pregnancy or observed in other populations during the COVID-19 pandemic [16]. These rates are consistent with other reports of elevated levels of depression and anxiety among pregnant individuals during the pandemic worldwide [17].

Many individuals who were pregnant during the COVID-19 pandemic are now in the postpartum period, a time in which new parenting and pandemic-related stressors are introduced. Since the previous history of depression and anxiety is one of the most robust risk factors in predicting postpartum depression [18, 19], the alarming rates of depression and anxiety among pregnant individuals during the pandemic [16] have put new mothers at heightened risk of mental health disorders. These mental health problems are associated with chronic physical conditions and low quality of life, and the overall cost of perinatal mental illness is large [20, 21]. Furthermore, COVID-19 has posed additional challenges to families, thereby increasing the support needs of mothers. Research suggests that since the onset of COVID-19, mothers with young children are experiencing heightened parenting stress due to deprivation of family support and altered family relationships [22]. For mothers with depression, these additional stressors could worsen the severity of their symptoms.

In line with the developmental origins of health and disease framework, ample evidence indicates that exposure to maternal depression in the first 5 years of life is linked to alterations in physiological regulation, cognitive impairments, and mental illness, with up to 60% of exposed children developing life course psychopathology [23, 24]. In early childhood, exposure to maternal mental illness predicts risk for children’s own mental health problems, developmental delays, atopic diseases, and injury risk [23–25]. Emerging research has raised significant concerns about the detrimental impact of COVID-19 on early child cognitive development. Infants who were born during the pandemic seem to have elevated risks for developmental delay in communication and personal-social functioning domains compared to infants born prior to the pandemic [26]. The intergenerational transmission of self-regulation model [27] suggests that maternal mental illness might impact child developmental outcomes through the self-regulation problems of mental illness on parenting abilities. Poor self-regulation can limit engagement in responsive parenting practices and lead to low supervision, poor planning, and harsh discipline, which in turn impede the development of children’s self-regulation [28]. Moreover, meta-analyses indicate that interventions that address both maternal (e.g., depression) and child (e.g., parenting) needs have larger effects than targeting either one alone [29].

Intervening early among mothers with depression is crucial in order to prevent long-term adverse consequences for mothers and their children. Without treatment, maternal depressive symptoms tend to remain consistent throughout the preschool years [8]. The negative consequences of maternal mental illness are most pronounced when depression persists [25, 30]. Untreated anxiety is also a concern as persistent maternal anxiety is associated with adverse child psychological outcomes, such as emotional and conduct problems [31, 32]. Although evidence-based treatments exist to address maternal mental illness, there are significant barriers to accessing care, and these barriers have been exacerbated by the COVID-19 pandemic. Prior to the pandemic, mothers in the perinatal period (i.e., pregnancy and the year following childbirth) identified lack of time and childcare as barriers to seeking mental health services [33]. These barriers may be particularly pertinent during the current COVID-19 pandemic due to overwhelming childcare demands, physical isolation from social and support networks, unemployment and financial strain, and closure of existing services [34–36].

Mothers with existing mental health problems in the perinatal period have identified web-based resources as a preference for additional mental health support [33]. Furthermore, peer support during the perinatal period is both protective against and associated with reductions in depressive symptoms [37, 38]. Mothers often rely on social support in the postnatal period to care for their infants, resume daily activities, and recover from childbirth [39]. Support from other mothers has been found to be particularly important in the recovery of postpartum depression and including peer support in the treatment of postpartum depression is associated with greater levels of treatment satisfaction [40].

Addressing the intergenerational impact of perinatal mental health problems requires innovative program design methods to simultaneously treat maternal mental illness and promote parenting abilities [25, 30]. In collaboration with a parent advisory board, we have developed the Building Emotion Awareness and Mental Health (BEAM) program to respond to the needs of mothers managing depression, anxiety, and parenting stress. The BEAM program provides app-based psychoeducation and group telehealth sessions with a mental health professional while prioritizing peer support as a central component of the program. Following the ORBIT model for developing behavioral therapies to treat or prevent chronic diseases and enhance health-promoting behaviors [41, 42], our research team previously conducted a phase II (preliminary testing) pilot trial of the BEAM program for mothers of children aged 18 to 36 months of age who self-reported moderate-to-severe symptoms of depression and or anxiety. The results revealed interaction effects with greater reductions in overall mental health problems among participants in the BEAM program compared to the control group [43]. Findings were used to determine the clinical impact of the program and inform improvements. The current study represents the phase III (efficacy testing) randomized controlled trial (RCT) of the BEAM program.

### Objectives {7}

The primary aim of this interventional study is to conduct an RCT to evaluate the efficacy of the BEAM app-based program for improving maternal mental health and parenting stress compared to a treatment as usual (TAU) control group. We hypothesize that participants who receive the BEAM intervention will report fewer depression symptoms (primary outcome) compared to the control group.

The secondary aims of the trial are to determine the benefits of BEAM for family relationships, parenting, and maternal and child socioemotional functioning. We hypothesize that participants who receive the BEAM intervention will report reduced anxiety symptoms, improved family relationship quality, more positive parenting skills, less parenting stress, and better child development outcomes. We will also explore whether changes in these outcomes occur as a function of treatment effects on maternal depression and parenting stress.

The current study will also examine exploratory outcomes of observed parent-child relationship quality, maternal sensitivity, and child emotion regulation via remote Zoom online assessments. Furthermore, exploratory outcomes will include physiological indices of wellbeing (e.g., sleep and daily activity) measured via Fitbits that participants will wear during the program. We hypothesize that participants who receive the BEAM intervention will display improved parent-child relationship quality, maternal sensitivity, and physiological health (e.g., improved sleep quality and increased daily activity) and that their children will demonstrate improved emotional regulation.

### Trial design {8}

A two-armed, parallel-design randomized controlled superiority trial with repeated measures will be used to evaluate the efficacy of the 10-week app-based BEAM intervention for mental health and parenting outcomes in parents of children aged 18–36 months (at study enrollment) compared to the treatment as usual (TAU) control group. Participants will be randomly allocated, using central randomization stratified based on telehealth session availability, in a 1:1 ratio to the BEAM program or TAU. Primary (depression) and secondary (anxiety, family relationship quality, parenting stress and behaviors, and maternal and child socioemotional functioning) outcomes will be assessed during the enrollment period (pre-test, T1), after the last week of the BEAM intervention (post-test, T2), and at follow-up (T3). Exploratory outcomes (parent-child relationship quality, maternal sensitivity, child emotional reactivity, and physiological indices) will be assessed at T1 and T2.

This trial adheres to the Standard Protocol Items: Recommendations for Intervention Trials (SPIRIT) guidelines [82] and was registered with ClinicalTrials.gov (NCT05306626). All procedures will be performed in accordance with the Research Ethics Board at the University of Manitoba, Fort Garry campus and the 1964 Helsinki Declaration and its later amendments. All participants will provide informed consent prior to enrollment in the study.

## Methods: participants, interventions, and outcomes

### Study setting {9}

Participants residing in Manitoba and Alberta will be recruited online. The intervention will be offered on an app-based psychoeducation platform with a private online forum, hosted on University of Manitoba servers. Assessments will be conducted online via the Research Electronic Data Capture (REDcap) tools for Personal Health Information Act (PHIA)-compliance and data security [44, 45].

### Eligibility criteria {10}

#### Inclusion and exclusion criteria

Eligibility includes identifying as a mother or female primary caregiver with a child aged 18–36 months; English speaking; above the age of 18; residing in Manitoba or Alberta, Canada; self-reported moderate-to-severe symptoms of depression and/or anxiety (above a clinical threshold) at the pre-test assessment that will occur prior to randomization; and consent to wearing a Fitbit. Eligible mothers will also need to be available to attend weekly telehealth sessions, complete the baseline Zoom assessment and/or Zoom orientation and complete the pre-test self-report assessment. Participants will also need access to an iOS or Android device. If a participant reports not having access to a device, they will be provided one to use for the duration of the study period. Eligible participants who consent to be randomized will be invited to participate in the study through email. In terms of exclusion criteria, mothers who report a history of attempted suicide in the past year or self-harm in the past 6 months will not be eligible for the current study as the BEAM program is not suitable to treat acute mental health needs. Instead, these participants will receive a list of mental health resources in their province that may be more appropriate.

#### Screening and enrollment

After informed consent is obtained, potential participants will automatically receive an online eligibility screener to complete. The eligibility screener will be used to confirm that they are 18 years or older, speak English, have at least one child between 18 and 36 months, and meet the threshold for moderate-to-severe (≥ 10) depression and/or anxiety using the Patient Health Questionnaire (PHQ-9) and the General Anxiety Disorder (GAD-7) scale; have access to an iOS or Android phone or tablet; are available to attend weekly telehealth sessions; and consent to wearing a Fitbit. Initial eligibility will be determined based on screener responses. Research coordinators will schedule a pre-test Zoom assessment (or Zoom check-in for those who are unable to complete the Zoom assessment) with individuals who meet eligibility based on the initial online eligibility screener. Participants will be considered eligible after they complete the screener questionnaire and the pre-test Zoom assessment or check-in and consent to randomization. Individuals who do not meet the eligibility criteria will be excluded from the RCT.

### Who will take informed consent? {26a}

Prospective participants are identified via online recruitment posters. Interested participants who click the study link will automatically be directed to an online informed consent form where they will read information explaining the study procedures in full detail before providing electronic written consent. Participants will have the option to contact the research coordinators through the BEAM program email address should any questions regarding the informed consent form arise.

### Additional consent provisions for collection and use of participant data and biological specimens {26b}

We will potentially conduct a longer term follow-up pending positive funding. If this follow-up occurs, further consent will be sought from all participants. No biological samples will be collected.

### Interventions

#### Explanation for the choice of comparators {6b}

The treatment as usual (TAU) control arm of the study is designed to account for the potential effects of time on depression symptoms [46]. The TAU group will continue to receive their standard care. The control group will be used to assess whether receiving the BEAM intervention is associated with improved outcomes (e.g., mental health, reduced parenting stress) compared to TAU.

#### Intervention description {11a}

The BEAM Program is based on best practices in telehealth and science-based program design principles aimed at promoting maternal mental health and supportive parenting. The BEAM Program will be delivered via a mobile application, which is designed and maintained by Tactica Interactive, managed by the research staff, and hosted on the University of Manitoba secure servers. The BEAM Program will also be delivered through weekly group telehealth sessions led by a mental health professional or trainee via the secure videoconferencing platform Zoom (Healthcare license). The BEAM application will include weekly 10–20 min asynchronous video modules on mental health and parenting as well as access to a private online forum for peer support from other participants and research staff.

An overview of the program materials is attached in the appendix (Appendix 1). Activities will be available each week where participants are encouraged to (a) reflect on the content and how it applies to their specific mental health and parenting situations and (b) engage in discussions related to the weekly topics with the community in the BEAM forum (described in detail below).

##### Psychoeducation

The BEAM program includes a psychoeducation component which will be composed of video modules on mental health and parenting delivered via the app. There will be at least one mental health video and one parenting video each week (approximately 10 min each).

Mental health videos will provide information and emotion regulation strategies for the transdiagnostic treatment of depression and anxiety disorders [47, 48]. The content draws principles from Unified Protocol (UP), a cognitive-behavioral therapy approach that addresses underlying features thought to contribute to emotional disorders (e.g., avoidance). Based on user feedback from the pilot study, self-compassion will also be a large focus of the mental health content given the link between self-compassion and psychopathology and the efficacy of self-compassion for improving a wide range of psychosocial outcomes [49, 50].

Supportive parenting videos will provide parents with emotion-focused parenting strategies that include information on child emotional development, sensitive responding, and behavior management strategies. These videos focus on helping parents understand their children’s challenging emotions and behaviors and focus on teaching parents to take a proactive approach in preventing negative interactions, tolerating extreme child emotions, encouraging compliant behaviors, and promoting a positive parent-child relationship.

##### Mood tracking

BEAM participants will complete brief weekly surveys responding to questions on depression, anxiety, parenting stress, positive mood, and recent stressful experiences. Participants will be provided with a weekly score for their depression, anxiety, and parenting stress. Participants will track and graph these weekly scores in a therapy notebook that is provided to them by the research team. Recent work analyzing mood-tracking within applications has found that apps with mood-tracking functionalities increase user self-awareness and understanding of emotions, making it an effective active therapeutic component [51].

##### Social support and community connection

Weekly group telehealth sessions will provide an opportunity for the clinical research team (i.e., those on the research team who have direct interactions with the participants) to check in with participants throughout the program. Participants will have the opportunity to discuss the material with their clinical coach (described below) and with other parents in the program, to ask questions about specific topics of interest, and increase a sense of community and social support.

Online Community Forum*:* The BEAM application also includes an online community forum designed to provide a space to reflect on skills learned in the intervention and access social support through connections with other mothers (participants and parent coaches; described below). All participants in the intervention group will participate in a closed online community forum that is moderated by a peer parent coach in consultation with our clinical research team of mental health professionals. The forum will consist of open-ended discussions between BEAM participants, peer advice, and sharing of anecdotes and/or non-identifying photos of their wellness journey. Furthermore, participants will have the ability to ask clinical coaches about specific mental health and parenting questions related to the program.

##### Coaching

Parent coaches will be mothers who have recently completed another research group-based intervention for their own mental health needs and who are interested in being a part of the community to promote the mental wellness of mothers. Parent coaches will help to conduct Zoom orientation meetings (detailed below) with participants prior to the program start date, will engage with participants on the forum, and co-facilitate weekly telehealth sessions.

Clinical coaches will be clinical trainees or registered mental health professionals. Clinical coaches will monitor the forums, facilitate weekly group telehealth sessions, and manage participant contact by email as needed. Clinical coaches will also participate in weekly consultation meetings with clinical supervisors (i.e., registered psychologists).

#### Criteria for discontinuing or modifying allocated interventions {11b}

Should participants disclose suicidal behavior, the clinical team will consult about these instances and advise on whether or not continued participation in the BEAM program is in the best interest of the participant. In accordance with Best Practice Guidelines for Telepsychology Services, clinical coaches may also decide to terminate a participant’s involvement in the e-health program if they deem it inappropriate for the participant to continue (e.g., if the participant cannot ensure confidentiality is maintained through access to a secure space and stable internet connection) [52]. Furthermore, participants may have discontinued access to the forum and may be denied access to the telehealth sessions if they engage in repeated violations of the terms of use (Appendix 2). In these cases, participants would still have access to psychoeducation materials and would still be invited to complete the assessments. The clinical coaches may also provide a referral to another provider or clinic if deemed appropriate.

#### Strategies to improve adherence to interventions {11c}

To promote engagement in the BEAM program, participants will be required to confirm availability for the Zoom telehealth sessions and complete the pre-test assessment (including the online survey and Zoom assessment) in order to be eligible for the study. Participants may opt out of the full Zoom assessment and complete a brief Zoom check-in instead if preferred. During the program, three scheduled weekly study-related notifications will be sent to participants in the intervention group through the BEAM app. Sending engagement reminders to participants has been found to contribute to greater mental health benefits from e-health interventions [53, 54] and increase the effectiveness of online parenting programs [55]. The proposed trial incorporates strategies such as reminders via email and telephone (SMS), which have also been shown to increase engagement [56]. Participants will receive a 1-page summary detailing the next steps in the BEAM program and will participate in a Zoom orientation meeting with a parent coach or clinical coach. The purpose of the orientation meetings is to welcome participants to the program, explain the program components, and answer questions, in an attempt to increase attendance and engagement in the program. Participants will be contacted by their clinical coach if they have not attended telehealth sessions for two weeks in a row. Parent coaches will also be assigned to facilitate and promote participant engagement on the forum.

#### Relevant concomitant care permitted or prohibited during the trial {11d}

Participants will be permitted to receive concomitant care for mental health problems, such as psychotherapy, medication, and/or support groups. No concomitant care and interventions are prohibited.

#### Provisions for post-trial care {30}

If further treatment is deemed necessary, a referral to another provider or clinic will be made, and a list of community services will be provided. No trial-related harms are expected.

### Outcomes {12}

#### Primary outcomes

The primary outcome is mean change in depression symptoms from pre- to post-intervention and from pre-intervention to follow-up, assessed using the Patient Health Questionnaire-9 (PHQ-9) [57], a self-report questionnaire that measures depression severity. Participant depression symptoms will be measured during eligibility screening pre-intervention (T1), immediately after intervention (T2), and at a 6-month follow-up (T3). The PHQ-9 includes nine items, asking participants to report over the past week how often they have been bothered by certain problems. Participants respond to each item using a 4-point Likert scale ranging from 0 (*not at all*) to 3 (*nearly every day*). Possible scores range from 0 to 27, with higher scores indicating more severe depression symptoms. Participant scores are summed, and a cutoff score of ≥ 10 will be used to identify participants who present with clinically concerning symptoms of depression [57]. The PHQ-9 cutoff score of 10 has been shown to demonstrate high sensitivity and specificity in detecting depression in a perinatal population [58].

#### Secondary outcomes

Secondary outcomes include mean change in maternal anxiety symptoms from pre- to post-intervention and from pre-intervention to follow-up assessed using the Generalized Anxiety Disorder 7-Item Scale (GAD-7) [59]. The GAD-7 is a self-administered scale with 7 items that are rated on a 4-point Likert scale (0 = *not at all* to 3 = *nearly every day*). A cutoff score of ≥ 10 will be used to identify participants who present with clinically concerning symptoms of anxiety. Scores range from 0 to 21, and participant scores are summed to obtain a total score. Higher scores indicate more severe GAD symptoms. The following suggested cutoff scores will be used: 10–14 (moderate anxiety) and 15 (severe anxiety). The mean change in parenting stress from pre- to post-intervention and from pre-intervention to follow-up will also be assessed using the Parenting Stress Index (PSI) [60], a self-report measure of parenting stress and interactional style. Scores are summed to obtain a total raw score ranging from 36 to 180, with higher scores indicating higher levels of parenting stress.

Additional secondary measures of parenting will be used to assess the mean change in family relationship quality across time points (T1 to T2 and T1 to T3). Parental assistance with child emotion regulation will be measured using the Parental Assistance with Child Emotion Regulation scale (PACER), a self-report measure of how parents use strategies to assist children with regulating their emotions [61]. Participant scores are summed to obtain a total score ranging from 50 to 350, with higher scores indicating greater engagement in strategies that support child emotion regulation. The mean change in parenting discipline practices will be assessed using the Parenting Scale, a self-report measure that assesses parenting behavior and dysfunctional discipline of parents with young children [62]. Scores are summed to create a total score ranging from 30 to 210, with higher scores indicating higher levels of ineffective discipline practices. Parenting behaviors will be measured using the Parenting Young Children (PARYC) measure, a self-report of parenting strategies and behaviors of caregivers with young children [63]. Scores will be summed, and the mean change in scores from pre- to post-intervention and from pre- to follow-up will be assessed. Scores range from 21 to 105, with higher scores indicating more proactive parenting behaviors.

Other secondary measures include co-parent relationship quality and support, additional mother socioemotional functioning measures related to mental illness, and child outcomes. The mean change in all measures will be assessed from pre- to post-intervention and pre-intervention to follow-up. Change in partner support across time points will be measured using the Social Support Effectiveness Questionnaire (SSEQ), a self-report measure of partner support effectiveness [64]. Scores are summed to obtain a total score with possible scores ranging from 0 to 80. Higher scores reflect more effective social support. Change in romantic relationship satisfaction will be measured using the 4-item Couple Satisfaction Index (CSI-4) [65]. The CSI-4 measures global evaluations of romantic relationships. Scores range from 0 to 21 with higher scores indicating higher levels of satisfaction. Anger will be measured using the Patient-Reported Outcomes Measurement Information System (PROMIS) Short Form, a self-report measure of frequency and severity of anger [66]. The mean change in anger scores will be assessed between T1 and T2 and T1 and T3. Total scores range from 5 to 25 with higher scores indicating more anger. Change in self-compassion across time points will be assessed using the Self-Compassion Scale Short Form (SCS-SF), a self-report measure of self-compassion [67]. Scores range from 12 to 60 with higher scores indicating higher self-compassion. Self-esteem will also be measured with the Single Item Self-Esteem Scale (SISE), a single-item measure that assesses global self-esteem [68]. Scores range from 1 to 7 with higher scores indicating higher self-esteem. Sleep problems will be measured using the PROMIS Sleep Disturbance Scale, a self-report measure of sleep disturbance [69]. Scores range from 8 to 40 with higher scores indicative of greater severity of sleep disturbance. Change in suicidal ideation severity will be measured using the Depression Symptom Index Suicidality Subscale (DSI-SS), a 4-item self-report measure of suicidal ideation [70]. Scores are summed to obtain a total score with possible scores ranging from 0 to 12. Higher scores indicate higher suicide ideation. The change in substance use (alcohol and cannabis) will be assessed using the Alcohol Use Disorder Identification Test (AUDIT) [71] and Cannabis Use Identification Test - Revised (CUDIT-R) [72], respectively. The AUDIT is a self-report measure of alcohol consumption, drinking behaviors, and alcohol related problems with scores ranging from 0 to 40. Higher scores are indicative of more hazardous alcohol use. The CUDIT-R is a self-report measure of cannabis misuse. Scores on this measure range from 0 to 32, with higher scores indicating more hazardous cannabis use. Scores of 12 or more indicate a possible cannabis use disorder. The mean change in child emotional and behavioral problems will be measured using the Preschool Pediatric Symptom Checklist (PPSC), an 18-item parent report measure of emotional and behavioral problems in young children [73]. Item scores are summed to obtain a total score ranging from 0 to 34, with higher scores indicating higher incidences of emotional and behavioral problems. Finally, the mean change in child temperament will be assessed using a subset of questions from the Early Child Behavior Questionnaire (ECBQ), a parent measure of temperament in children aged 1 to 3 [74]. Scores range from 1 to 7 with higher scores indicating higher levels of the temperamental trait.

#### Exploratory outcomes

Exploratory outcomes include changes in parent-child relationship quality, sensitive parenting, and child emotional reactivity, using observational measures obtained during a remote Zoom video assessment with mothers and their children that will occur at pre- and post-intervention. Participants will also be asked to wear a Fitbit Inspire 2 throughout the trial. Various types of physiological data will be collected (i.e., heart rate, sleep quality and duration, and daily physical activity) at pre- and post-intervention in order to measure changes in heart rate, sleep duration, sleep quality, and physical activity. The Fitabase software will be used to manage data from participants’ Fitbits as Fitabase allows researchers to amalgamate data from across participant Fitbits [75]. The feasibility of the BEAM program will be assessed at post-intervention using the mHealth App Usability Questionnaire (MAUQ) [76] and other questionnaires developed for the BEAM program. Engagement and adherence linked to user IDs will be tracked from back-end app data using Firebase and Google Analytics, such as number of logins and time spent on the app, forum posts, and telehealth session attendance. A final exploratory outcome will involve analyzing the qualitative program data which will include the content of posts and comments within the forum and feedback from participants after the program completion (post-intervention) for program improvement purposes.

#### Descriptive measures

Descriptive data will be collected via survey questions on demographic and socioeconomic characteristics at pre-intervention. Items will include education level (high school degree or lower vs. post-secondary education), age, household income, changes in household income due to COVID-19, marital status (married or in a domestic partnership vs. other), number and ages of all children, type of community in which one lives (large city vs rural area), and medications. A measure of personality using the Big Five Inventory-10 (BFI-10), which will be adapted from the original questionnaire [77], will be administered at pre-intervention. Participants will also be asked about other treatments and service use throughout the duration of the BEAM intervention.

#### Participant timeline {13}

**Fig. 1:**
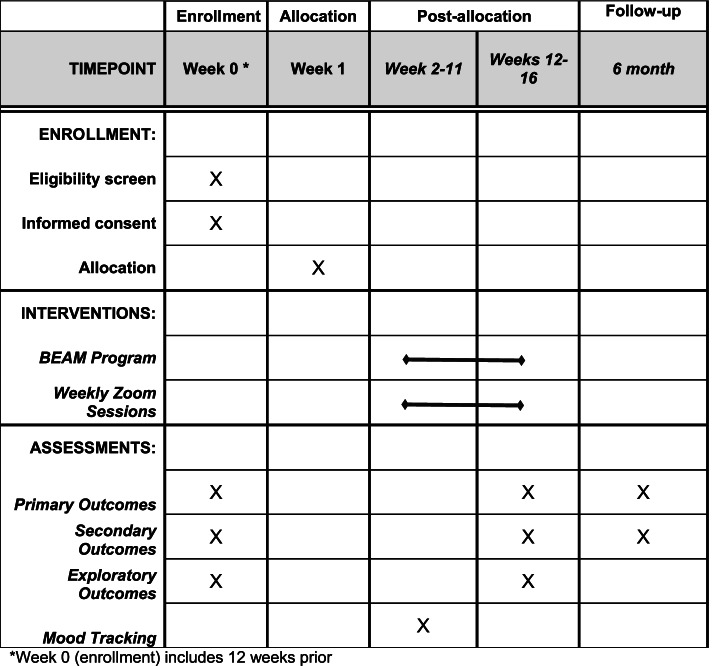
SPIRIT schedule of enrollment, intervention, and assessments

The overall participant timeline is illustrated in Fig. 1.

Week 0 (T1): Eligible participants who consent to randomization and to participate in the program will receive notice of enrollment. Participants will participate in a clinical orientation meeting with a member of the clinical research team (i.e., a parent or clinical coach) and complete the pre-test assessments, including primary, secondary, and exploratory outcome measures.

Week 1: Participants randomized to the experimental condition will receive an email regarding login and account information. Program information will be sent electronically to participants from the study team, and participants will watch introductory videos to become familiar with the app. Participants will begin engaging in the forum.

Weeks 2–11: Participants will watch weekly videos and will complete mood tracking questions on depression and anxiety symptoms, parenting stress, recent stressful experiences, and positive mood for self-monitoring purposes. Participants will also participate in weekly Zoom telehealth group sessions and continue engaging in the forum.

Weeks 12–16 (post, T2): Participants will complete a full post-test assessment of primary, secondary, and exploratory outcome measures.

Month 6 (follow-up, T3): Participants will complete a follow-up assessment of primary and secondary outcome measures.

#### Sample size {14}

Based on the findings from a meta-analysis, digital interventions for depression that include a human therapeutic guidance component (as is the case with the BEAM program group therapy sessions) have a moderate effect on depression (*g* = .63) [78]. Thus, an a priori, one-tailed, two-group power analysis was conducted using a moderate effect size (*d* = .5) using the G* Power 3 software [79]. The power analysis indicated that we would need 51 participants per group to reach 80% power (*α* = .05) to detect a clinically significant change in depression. To account for anticipated attrition of approximately 25%, a total sample of 140 with 70 in each group (intervention and control) will be the target for recruitment.

#### Recruitment {15}

Participants in Alberta and Manitoba, Canada, will be recruited online via multiple sources such as advertisements on social media platforms including the Hearts and Minds Research Lab Facebook, Twitter, and Instagram accounts. Recruitment materials will also be emailed to relevant community agencies and daycares. Individuals will respond to program advertisements by clicking a link to the online eligibility screener. Individuals who meet all the eligibility criteria and consent to randomization will be invited to participate in the study. The recruitment duration period will last 3 months, and recruitment will be monitored by the research staff. Participants will be eligible to receive up to $150 Canadian (CAD) compensation for their enrollment and participation for the entire study duration. There are no incentives provided to trial investigators for participant enrollment.

### Assignment of interventions: allocation

#### Sequence generation {16a}

Allocation will be conducted using a randomized intervention model. Participants will be randomly allocated in a 1:1 ratio to the BEAM program or TAU. Randomization will be stratified based on which telehealth group timeslot participants identified being available for. Central randomization, by computer-generated sequencing using an online tool (https://www.randomlists.com/team-generator), will be conducted by a research assistant that is not affiliated with the BEAM clinical research team.

#### Concealment mechanism {16b}

Central randomization using computer-generated sequencing will be conducted by a research assistant who is not involved in clinical care (i.e., group therapy sessions, forum engagement) or pre- and post-test assessments to ensure allocation concealment.

#### Implementation {16c}

A research staff member who is not associated with the study will conduct the randomization and sequence generation. After the completion of the pre-test assessment, a research assistant will enroll participants in the trial, check the computer-generated assignment and inform participants of their enrollment and condition in the trial.

### Assignment of interventions: blinding

#### Who will be blinded {17a}

Participants, clinical coaches, and parent coaches will not be blinded to condition. Data analysts will not be blinded to conditions either as the analysis will be conducted by the research team. The trial did not have the funding for separate statisticians. The research assistants responsible for coding observational measures in assessments will be blinded to participants’ group assignments.

#### Procedure for unblinding if needed {17b}

Not applicable as participants, clinical coaches, and parent coaches will not be blinded to condition.

### Data collection and management

#### Plans for assessment and collection of outcomes {18a}

Data will be collected via self-report measures, Fitbit devices (Fitbit Inspire 2), online video assessment, the use of back-end app data via Firebase and Google Analytics, and forum content.

The primary outcome of change in depression symptoms will be assessed using the Patient Health Questionnaire (PHQ-9) [57]. Secondary outcomes include changes in *anxiety symptoms*, assessed using the Generalized Anxiety Disorder 7-Item Scale (GAD-7) [59], *family relationship quality*, assessed with the Parental Assistance with Child Emotion Regulation (PACER) [61], the Social Support Effectiveness Questionnaire (SSEQ) [64], and the Couple Satisfaction Index (CSI) [65], *parenting*, assessed using the Parenting Stress Index (PSI) [60], the Parenting Scale [62], and the Parenting Young Children (PARYC) measure [63], and *maternal functioning* assessed using a variety of validated self-report questionnaires for mental health symptoms, including the Patient-Reported Outcomes Measurement Information System (PROMIS) Anger Subscale [66], the Self-Compassion Scale Short-Form [67], the PROMIS Sleep Disturbance Scale [69], the Depression Symptom Index Suicidality Subscale [70], the Single Item Self-Esteem Scale [68], the Alcohol Use Disorder Identification Test (AUDIT) [71], and the Cannabis Use Disorder Identification Test – Revised (CUDIT-R) [72]. There will also be additional measures of suicidality and self-harm, depression persistence, recent stressful experiences, mental health medication and service use, and an additional measure of substance use, which have been developed for the BEAM project. *Child Functioning* will be measured using validated questionnaires for child mental health and behavior, including the Preschool Pediatric Symptom Checklist [73] and a subset of questions from the Early Child Behavior Questionnaire [74]. Additional measures of screen time and child health, which have been developed for the BEAM program, will also be used.

Online video assessments will be conducted to measure exploratory outcomes of parent-child relationship quality, child emotional reactivity, maternal sensitivity (e.g., Maternal Q-sort), and sensitive parenting (e.g., child-directed speech). Online video assessments will take place over Zoom with mother-child dyads. Assessors will be undergraduate psychology students who have undergone extensive training in conducting Zoom assessments with families. Training involves watching practice videos and practicing the assessments with pilot families under the supervision of psychology graduate students as well as registered psychologists on the research team. Exploratory outcomes will also include physiological measures obtained through the use of Fitbit devices (Fitbit Inspire 2), including heart rate, sleep quality and duration, and physical activity. Feasibility of the BEAM program will be assessed using the mHealth App Usability Questionnaire self-report questionnaire (MAUQ) [76] and other questionnaires developed for the BEAM program. Firebase and Google Analytics will be used to track program engagement and adherence and the content of posts and comments within the forum will be used for program improvement purposes.

Additional self-report questions will be administered to assess demographic and socioeconomic characteristics and personality will be assessed using the Big Five Inventory-10 (BFI-10) measure, which will be adapted from the original questionnaire [77].

#### Plans to promote participant retention and complete follow-up {18b}

Three scheduled study-related reminders will be sent to participants each week through the BEAM app and participants will receive one weekly email reminder which will include information for group telehealth sessions. Eligible participants will receive up to $150 Canadian (CAD) compensation for participating for the entire duration of the study. Participants will receive an honorarium of $20 CAD for completing the pre-assessment questionnaires, $30 CAD for completing the post-intervention assessment, $20 CAD for completing the follow-up assessment, and $30 CAD if they complete all weekly surveys ($3 CAD per week) and will receive another $50 CAD if they choose to mail back the Fitbit used for data collection during the study. Furthermore, the clinical research team will have weekly meetings to monitor the adherence and retention of participants. For instance, group attendance will be discussed and additional email check-ins with participants will be performed when advised by the registered psychologists on the team.

#### Data management {19}

REDCap is managed by The George and Fay Yee Centre for Healthcare Innovation, which is a hired consultant on the proposed project and will support secure data collection and management. RCT data will be stored on a secure server in accordance with the University of Calgary’s Data Retention Policy and the University of Manitoba’s PHIA policies. Once data is downloaded, all identifying information will be removed, which aligns with the University of Manitoba’s REDCap policies. All patient health information and identifiable information will be kept for up to 5 years. Birth dates will be converted into age in months, and birthdate information will then be destroyed. For the storage of Zoom assessment data, raw video data will be destroyed no later than 5 years after collection. Once videos have been coded by trained observers, the original file and all copies will be deleted. Measures to promote the quality of data entry will include range checks for all data values entered and having three research assistants review and ensure correct data entry in REDCap.

#### Confidentiality {27}

The BEAM program will occur on a secure online platform and participant confidentiality will be protected throughout all phases of the study in accordance with University of Manitoba and University of Calgary ethics guidelines. Only the research team will have access to study data and only de-identified data will be transmitted to coinvestigators. All those with access to data are trained in the University of Manitoba’s and University of Calgary’s ethics and data safety protocols, have completed additional training on the Public Health Information Act, and have taken an oath of confidentiality.

Assessment data will be stored securely on REDCap or on password-protected University of Manitoba secure data servers. Any documents including identifying information will be password-protected and stored on University of Manitoba secure data servers. All questionnaire data will be exclusively linked to de-identified participant ID numbers once it has been downloaded from REDCap. Qualitative data from forums will be anonymous and not linked to any identifying information on the platform. Fitbit data will be stored in Fitabase’s high-security cloud-based database [75] until the trial is complete, at which point it will be downloaded and deleted.

Precautions will be taken to manage any risks to confidentiality of personal data on the forum. These precautions include a Risk Management Protocol, Data Management Protocol, participant agreed upon Terms of Use, and the ability for program coaches to remove any posts that violate the terms or use and to email any participants should there be concerns.

To protect participant privacy and confidentiality and to ensure that no health information is shared on the forum, all participants will be required to agree to the community guidelines including the Rules and Terms of Use of the online forum and will agree not to disclose any identifying information to other participants. Security measures include monitoring of the forums by the study team, and the ability for other participants to anonymously flag posts for review. A risk management protocol will be put in place by clinician leads in each province to manage any potential crisis-related mental health or child safety needs.

During the weekly telehealth sessions, participant anonymity cannot be guaranteed. The telehealth sessions will be hosted on a secure Zoom Healthcare account and will be password-protected. The clinical coaches will outline the limitations of confidentiality and anonymity at the beginning of the first telehealth session. Telehealth meetings will not be recorded or included in any assessment; however, attendance will be tracked. The Jane app will also be used as the clinical record management tool (i.e., to track attendance to telehealth sessions and make session notes).

Participants will become familiar with the limitations to confidentiality and of the measures that will be taken to protect the confidentiality during the informed consent process before beginning in the study.

#### Plans for collection, laboratory evaluation, and storage of biological specimens for genetic or molecular analysis in this trial/future use {33}

Not applicable, no biological specimens will be collected.

## Statistical methods

### Statistical methods for primary and secondary outcomes {20a}

Between-group differences in primary and secondary outcome variables, as well as quantitative exploratory outcome variables (observational and Fitbit data), will be analyzed using linear mixed models. Models will test both a linear and quadratic time coefficient, where quadratic time coefficients will be removed from final models if not significant. An *α* of 0.05 will be used to determine statistical significance. Effect sizes of 0.20, 0.50, and 0.80 will be interpreted as indicating small, medium, and large effects, respectively [80]. Standardized effect sizes for linear mixed models will be derived based on existing recommendations for linear mixed models [80, 81]. The research team will also investigate if there is baseline moderation by symptom severity to examine whether the BEAM intervention is more effective than the control group for participants who have higher symptom levels at pre-intervention (e.g., depression, anxiety, insomnia).

Exploratory qualitative data will be analyzed thematically [82]. First, two analysts will concurrently, but independently read the transcripts for surface descriptive content, and organize like-with-like ideas. Second, transcripts will be re-read for latent meaning to better understand participants’ meaning, including looking for contradictory or confirmatory statements. Each analyst will independently generate and systematically apply themes across all transcripts. Semantic validity checks will ensure different words and phrases within a category have similar meanings [82]. To further enhance rigor, constructs will be deemed saturated (akin to statistical significance in quantitative work) when no new or additional constructs are identified, consensus is reached on all overarching themes, and no alternative explanations were found with subsequent interviews [82].

### Interim analyses {21b}

No interim analyses are planned.

### Methods for additional analyses (e.g., subgroup analyses) {20b}

Subgroup analyses will be conducted to explore the differences in outcomes for those who do and do not attend telehealth sessions regularly. Additional subgroup analyses may be explored based on emergent research questions of interest.

### Methods in analysis to handle protocol non-adherence and any statistical methods to handle missing data {20c}

Analyses will be conducted on an intent-to-treat (ITT) basis [83]. Regardless of participants’ adherence to the intervention or whether they withdrew from the study, data for every participant who was randomized to either the control or intervention group will be included in ITT analyses. Analyses that include only participants who completed the intervention will also be conducted for comparison purposes. Missing data will be handled using maximum likelihood [84], which estimates values based on all available data and thus produces unbiased model parameters [85].

### Plans to give access to the full protocol, participant-level data, and statistical code {31c}

The full trial protocol will be made publicly available through BMC Trials. Dr. Tomfohr-Madsen and Dr. Leslie Roos will remain the custodians of the data, as the primary investigators. Data will be analyzed independently and will not be released to any third party (including the funder) before the trial is completed. In accordance with the International Committee of Medical Journal Editor’s guidelines, the authors will share de-identified patient data that underlies results presented in reports no later than 6 months after publication. The primary investigators will ensure data is available upon request.

### Oversight and monitoring

#### Composition of the coordinating center and trial steering committee {5d}

Dr. Tomfohr-Madsen and Dr. Roos, as the primary investigators, will be responsible for the overall management of the project. Dr. Tomfohr-Madsen has expertise in program development, intergenerational transmission of stress, and clinical trials and will be the clinical lead in the preparation and delivery of mental health psychoeducation content. Dr. Roos has expertise in maternal-child health interventions and will lead platform development and parenting content for participants. Dr. Reynolds is a clinical psychologist with expertise in maternal mental health, program evaluation, knowledge translation, and mixed-methods data analysis. Dr. Reynolds will assist with exploratory qualitative analyses and integrating participant feedback into specific revisions to existing protocols. Dr. Sauer-Zavala is a co-developer of the Unified Protocol and has experience with efficacy testing for online interventions. Dr. Giuliano will lead cardiac and cognitive data processing teams. Dr. Soderstrom focuses on language development across cultures and contexts and examines conversational interactions between caregivers and infants. Dr. Protudjer has expertise in mixed methods and will co-lead exploratory qualitative data analysis to develop content for patient-identified needs.

#### Composition of the data monitoring committee, its role, and reporting structure {21a}

Study investigators will monitor the trial. There will be weekly meetings with research assistants and the primary investigators (i.e., Dr. Tomfohr-Madsen and Dr. Roos) to discuss and review each phase of the trial (e.g., recruitment, randomization, and data collection). There will also be weekly meetings with the clinical coaches and tech facilitators (i.e., those who are interacting with research participants) and primary investigators to discuss and resolve all potential questions and concerns of participants. Given the minimal risks of the program, short duration (10 weeks of intervention), and relatively small sample size, the current trial does not require a formal data monitoring committee.

#### Adverse event reporting and harms {22}

No known risks to mothers are associated with digital psychoeducational interventions. Should participants report significant distress or request additional support, clinical coaches follow up with these participants and consult with clinical supervisors using a risk management protocol. The primary investigators will check in with clinical coaches and parent coaches regularly to ensure that the study participants’ questions and concerns are responded to appropriately. No adverse events were reported in our pilot study.

We will follow the University of Manitoba’s standard procedures for reporting adverse events and protocol violations/deviations to the Research Ethics Office at the Fort Garry Campus and privacy breaches to the Access and Privacy Office.

#### Frequency and plans for auditing trial conduct {23}

The Research Ethics Board may request an audit. However, the study team has no plans for independent auditing of trial conduct.

#### Plans for communicating important protocol amendments to relevant parties (e.g., trial participants, ethical committees) {25}

Any amendments to the protocol will be submitted to the Ethics Board at the University of Manitoba, who will review and approve the amendments. If the amendment requires that revised information be communicated with enrolled research participants, this will be done through a consent addendum, which will be provided to participants through email.

#### Dissemination plans {31a}

Research findings will be disseminated to academic and nonacademic audiences, regardless of the magnitude or direction of the effect, within 12 months of trial completion. We will prepare several articles describing the effects of the BEAM program on parenting stress, mental health, and child well-being for publication in peer-reviewed journals. Papers will also be posted rapidly on preprint servers and will be open access to allow for wider reach. The principal investigators and trainees on the research team will present findings at national (e.g., Canadian National Perinatal Research meeting) and international (e.g., Society for Research in Child Development) conferences. Dissemination of infographics and bimonthly reports will occur through our partnerships with community agencies and existing knowledge users in government (e.g., Leanne Boyd, director of policy development, research, and evaluation, Healthy Child Manitoba). Research findings will be shared in lay summaries on lab and study websites and social media pages. We will work with media relations at the University of Calgary and our networks to disseminate research findings rapidly through local and national media. Furthermore, if trial findings substantiate the efficacy of the BEAM program we will explore opportunities for health system integration through scale-up funding.

## Discussion

Digital mental health and parenting interventions have the potential to (1) address the alarming rates of maternal mental health concerns during the COVID-19 pandemic, (2) help to protect against the adverse intergenerational consequences on child development, and (3) provide easily accessible programming to mothers who are in need of mental health and parenting supports, both during the COVID-19 pandemic and beyond. The BEAM program is a newly developed evidence-based app program that prioritizes social connection and provides psychoeducation and weekly telehealth sessions with mental health professionals. This RCT will build on a pilot trial of the intervention and provide further efficacy testing of this treatment for mothers experiencing depression and/or anxiety. Investing in maternal mental health early is expected to yield high health and economic benefits by preventing the long-term consequences of maternal depression from becoming imbedded in children’s biological and behavioral development [25]. Findings from this RCT will improve our understanding of mental health and parenting among mothers with young children and in turn help to improve maternal and child health and family well-being.

## Trial status

Recruitment began in mid-November 2021 and finished in February 2022. The date of first enrollment was November 17. The intervention was launched at the end of February 2022 (with estimated completion in October 2022 for the collection of all primary outcomes).

## Data Availability

Data will be maintained in a secure database that only the study investigators will have access to.

## Appendix 2

### Forum terms of use

By joining, we ask that all clients agree to not post the following information. Posting on these topics may be upsetting to other participants or violate University of Manitoba data agreements. Should you need to discuss any of these areas with your clinical coach, you are welcome to contact them via email.

By clicking this box, I agree to refrain from posting on the following topics:

1. Personal information that could identify you to group members, including:
  - Address
  - Phone number
  - Other personal/demographic information
2. Substance use or self-harm or suicidal behaviors
  - If you are experiencing any of the following, please email your please clinical coach, who will provide further support.
    i. Distressing suicidal thoughts, plans or are actively self-harming
    ii. Alcohol, marijuana, or substance use that is impacting your daily function and you are having difficulty controlling.
3. Information relating to partner abuse:
  - If you are experiencing any of the following, please email your clinical coach, who will provide further support.
    i. Partner abuse, experience of maltreatment or endangerment from a partner.
4. Personal health information including:
  - Medical diagnoses
  - Test and laboratory results
  - Insurance information
  - Mental health diagnoses
5. Spam/advertising/self-promotion in the forums
  - These forums define spam as unsolicited advertisements for goods or services.
6. Copyright-infringing materials
  - Providing or asking for information on copyrighted materials is forbidden.
7. Offensive posts, links, or images
  - Any materials that constitute defamation, harassment, or abuse
  - Material that is sexually or otherwise obscene, racist or otherwise discriminatory
8. Disrespectful comments towards other group members
  - All posts should be respectful and courteous.
  - Any instance of threatening, harassing, or discriminatory behavior is grounds for you to be denied access to the forum.
9. Posts including misinformation such as:
  - Deceptive information
  - False rumors
  - Propaganda

## Appendix 3

### Participant informed consent form

**Informed Consent Form – BEAM: Building Emotion Awareness & Mental Health**

Principal investigator:

Dr. Leslie E. Roos, Duff Roblin Building, Department of Psychology, University of Manitoba

Leslie.Roos@umanitoba.ca

This consent form should give you the basic idea of what the research is about and what your participation will involve. If you would like more detail about something mentioned here, or information not included here, feel free to ask. Please take the time to read this carefully and to understand any accompanying information.

**About this study:**

You and your child are invited to participate in a clinical trial study in which researchers will be recruiting mothers and their 18–36-month-old children in Manitoba and Alberta. Our goal is to support maternal mental health and reduce parenting stress using an interactive psychoeducation and social connection mobile app.

***Randomization***

Eligible participants will be randomized, which means that you will be put into a group by chance, like flipping a coin. You will have an equal chance of being placed in either the intervention or control group.

*Intervention group:* If you are randomized into the intervention group, you will be invited to participate in a 10-week online mental health and community support intervention called the BEAM Program (Building Emotion Awareness & Mental Health) that includes educational materials on improving your mental health and behavior management strategies for 18–36-month-old children. You will also have access to a closed forum where you and other mothers can communicate for social support. You will also be asked to complete questionnaires at varying time points throughout the study (described below).

*Control group:* If you are randomized into the control group, you will be invited to complete online questionnaires at varying timepoints throughout the study (described below).

**Who is conducting this study?**

This study is being conducted by Leslie Roos, Ph.D. (principal investigator) and additional co-investigators and the University of Manitoba and University of Calgary (Lianne Tomofohr-Madsen, Ph.D.; Shannon Saur-Zavala, Ph.D.; Jennifer Protudjer, Ph.D.; Ryan Giuliano, Ph.D.; Gerald Giesbrecht, Ph.D; Catherine Lebel, Ph.D.; and Melanie Soderstrom, Ph.D). It has been funded by Research Manitoba.

**Why am I being asked to participate in this part of the study?**

You are being asked to participate in this study because (1) you are a mother with a 18–36-month-old child, (2) you described experiencing symptoms of depression on the online survey, and (3) you expressed interest in our study. Your participation will not affect your relationship with the University of Manitoba, the University of Calgary, or any mental health or social services you are receiving now or would like to receive in the future.

**What will I do if I choose to participate?**

If you choose to participate, you will complete our online assessment and be randomized into either the intervention or the control group.

**Weekly assessments** will be conducted, starting before the first session (week 0) and continuing for 10 weeks, with a follow-up assessment at week 18. Full 45-min assessments of anxiety, emotion dysregulation, parenting stress, and health care needs will occur at week 0, week 10, and follow-up (week 18). All assessments will be conducted online via the REDcap interface for PHIA compliance, to protect your information and ensure confidentiality.

***Intervention group***

If you are placed in the intervention group, you will have access to an online platform where clinical topic experts will provide multiple short weekly 5–20-min videos, on mental health and parenting topics that focus on emotion regulation and supportive family relationships. The BEAM platform is designed using a best-practice treatment that is tailored to your individual needs and is responsive to your feedback. You will also be asked to participate in an online psychoeducation platform where you will share your experiences: managing stress, mental wellness, and parenting.

Mental health psychoeducation will focus on the Unified Protocol model of understanding, managing, and regulating emotions as a path to mental wellness. Parenting attention will be focused on reducing common challenges (e.g., bedtime, sharing with siblings, screen time limits) and increasing activities that support child development (e.g., bonding, routines, impulse control, managing big emotions). We will also invite you to ask questions of family wellness experts across topics such as partner conflict and pediatric care, who will provide responses visible to all forum members.

You will be guided through the 10-week program with a community of other mothers, while receiving both clinical and peer coaching support. The BEAM program also includes a **closed online community forum**. The forums will be personalized but confidential with 20–40 parents who will move through 10 weeks of content together. This forum will include conduct guidelines and will be mediated by a mental health clinician (MA or PhD in psychology).

Should you be randomized to the intervention group, you will be contacted by your clinical coach to request your phone number and address prior to starting the program. This information would only be used by the clinical team to contact you in case of crisis-related clinical concerns or to problem solve and challenges with the App and is consistent with safety precautions assumed by the licensed clinical psychologists managing the team. Any emails will be deleted once problems are resolved and no records of phone calls or emails will be considered research data. The clinical team will not have access to questionnaire data linked to your name or email.

***Control group***

If you are placed in the control group, you will participate in the online assessments described above and also receive a list of community resources in your province.

Participation in both the intervention and control groups will be compensated up to $70, based on participation in assessments, described below.

**What if I no longer want to participate?**

If for any reason, you are unable to or do not wish to participate in the assessments or treatment, you have the right to do this. There will be no penalty, except that you will no longer be able to participate in the BEAM program. Should you choose to discontinue your participation, it will in no way affect your relationship with the Hearts and Minds Lab, the University of Manitoba, or the University of Calgary or affect any services that you may be receiving or will receive in the future. We would offer referrals to other services if you request. If you ever want to withdraw, you can contact the project coordinator at research@heartsandmindslab.com with BEAM program withdrawal in the subject line.

**How much time does it take to participate?**

If you are placed in the intervention group, clinical topic experts will provide weekly videos that range from 5 to 20 min long and also include digital handouts for you to complete. Weekly assessments in both the intervention and control groups will take 5 min to complete. Full 45-min assessments of mental health, parenting, family wellness, and health care needs will occur at week 0, week 10, and follow-up (week 18).

We will ask all participants to log onto the platform at least weekly and to spend at least 30 min watching relevant videos and 30 min engaging in the online forums. The online nature of the platform allows you to personalize how much additional time you want to spend on the platform based on your schedule and mental health/parenting needs.

We hope that you incorporate the skills you learn into your daily activities. Fully participating and practicing mental health and parenting skills is linked to improvements in well-being, but the extent of participation and practice is up to you. If you have not been participating on the app, one of your coaches may reach out to ask about your wellness and see if there is anything else that we can do to support you.

**How will your privacy (or confidentiality) be protected?**

First and foremost, you do not have to answer any question that you do not feel comfortable answering. Because your honest answers are so valuable to us, we will do everything we can to keep everything about your child and you confidential (e.g., completely private). Here is how we will protect your privacy:

1. The BEAM program will occur in a secure online platform designed that is designed for you to remain anonymous to other users. Only the research team of co-investigators as well as supervised undergraduate and graduate students trained in the University of Manitoba’s and University of Calgary’s ethics and data safety protocols may have access. Your data will be stored on a protected secure server.
2. Assessments will be collected on REDcap, a secure web-based application designed exclusively to support data capture for research studies and linked to a de-identified participant number. Any identifying information will be stored separate from your data and only in the secure research environment hosted on REDcap.
3. All those with access to data have completed TCPS Core Ethics training and additional training on the Public Health Information Act to ensure confidentiality. All researchers involved in the project have completed their PHIA and TCPS certificates and Oath of Confidentiality.
4. We will write papers and make presentations using the information from this project for scientific, policy, or community knowledge purposes, and we will never use names or information that could identify anyone in the study.
5. All copies of identifiable data collected (e.g., videos, names) will be destroyed within 5 years after collection.
6. De-identified data (e.g., standardized questionnaire responses, aggregated program use data, sociodemographics linked to an ID number) may be made available on public data platforms such as open science framework or a requirement by a granting agency or journal. Any information sent out of the University of Manitoba will not show your name or address, or any other identifiable personal information about you. However, despite efforts to keep your personal information confidential, absolute confidentiality cannot be guaranteed. Your personal information may be disclosed if required by law.

**Are there any times when your child’s and your information could be shared with others?**

Yes. These are called “exceptions to confidentiality.” All research team members will be trained to follow mandatory reporting regulations that require university employees/staff to report suspected cases of child maltreatment (abuse, neglect, or endangerment). The BEAM program does not explicitly focus on child maltreatment. However, should you volunteer information over the course of treatment that leads us to suspect that your child has experienced or is experiencing maltreatment, we may ask you additional clarifying questions and may be obligated to contact a child welfare agency.

We will also take precautions to determine your safety and the safety of your child or others if we hear that you or your child plans to hurt him/herself or someone else. This may mean notifying others. During treatment, you may disclose that you are having thoughts or urges related to self-harm or have thoughts about death. We will ask some follow-up questions to learn if these are recent and if the behavior is high-risk. If we believe these behaviors are life-threatening, we will take necessary actions to prevent this from happening, such as calling a local mobile crisis unit that operates through the police station and will come to the lab to assist you. We want to emphasize that telling us you engage in self-harm or have had recent thoughts about death will not automatically result in our calling a local crisis unit.

Finally, there are other times we may share information with others, but this will only be done with your consent. For example, if you have another provider that we should contact to discuss treatment, we will have you complete a standard release of information form. This is not considered part of your BEAM treatment data. In addition, we will have you complete a form of emergency contacts. We would only use these contacts if we lost contact with you while you were part of treatment and were concerned about your well-being.

**Will we ever ask you to stop participating?**

If we are concerned that you are distressed while participating in the project, we may ask that you take a break and consider ending the current session and potentially rejoin at a different time. We may also ask that you consider ending your participation in BEAM and refer you to a higher level of care if you experience worsening symptoms over time (i.e., become suicidal with an active plan).

**What are the possible risks to you as a participant in treatment?**

Being asked questions about your emotions and experiences can sometimes be distressing and it is possible you may experience intense emotions during treatment. You do not have to answer any question that you do not feel comfortable answering. The other risk is “exceptions to confidentiality” and the possibility that if you report being a danger to yourself, or others, or if it is suspected that your child has been or is being maltreated, we may need to contact authorities.

**What are the potential benefits to you as a participant?**

*The following information applies to those who are randomized into the intervention group.*

The Unified Protocol has been shown to help people who may have difficulties regulating emotions, as well as being an effective treatment for depression and anxiety. Research has shown that persons report better emotion regulation after completing. The parenting skills material that you will receive is similar to other programs that have shown that receiving this information can help lower parenting stress and prevent the development of children’s behavioral or emotional problems. However, because we are presenting the material in a new way on this online platform, as part of the clinical research, we do not know yet if these parenting skills will have effects on you or your child.

You will also have access to a community support forum consisting of ~ 30 parents to promote social connection. You will also be in contact with trained clinicians and peer coaches that will guide you through the use of the online platform and provide answers to questions, as well as opt-in videos on specific topics like insomnia or domestic conflict so that you may personalize the BEAM program for your own family needs.

As it applies to all participants, some people may find it interesting to be a part of a research project. Finally, the information we learn from this project will benefit society’s understanding of the links between the mother’s mental health and child development.

**Will you be compensated for your participation?**

Yes, in participating in this study, you will be compensated $20 for completing the pre-assessment questionnaires and zoom assessment (week 0), $30 for completing post-assessments (week 10), and $20 for completing the follow-up assessment (week 18). You will also receive $30 for completing all weekly assessments (or $3 for each weekly assessment you complete). Finally, at the end of the program, you can decide to keep the Fitbit we send you or mail it back for an additional $50. Therefore, you can receive a total of $150 for participation in the entire study. There is no additional compensation for participation in the intervention group; however, the treatment will be provided to you by skilled clinicians at no additional cost to you.

**What will be done with the results of this project?**

We will study the data for the whole group and not for any one individual. We may share the results in psychological and educational presentations, books, and articles. In addition, the data collected will be used in students from the lab’s Honours, Master’s, and Ph.D theses/dissertations. We will not use your name or your identity in any presentations or publications.

**If you have questions**

If you have any questions, please feel free to contact the principal investigator Dr. Leslie Roos at leslie.roos@umanitoba.ca or the project coordinator at research@heartsandmindslab.ca. Please include BEAM Program Question in the subject line.

Your personal health information is being collected under the authority of the University of Manitoba Act. The information you provide will be used by the university to provide health care services at University Health Services. Your personal health information will not be used or disclosed for other purposes, unless permitted by the Personal Health Information Act (PHIA). By clicking “Agree” below, you have indicated that you have understood to your satisfaction the information regarding participation in the research project and agree to participate as a subject. In no way does this waive your legal rights nor release the researchers, sponsors, or involved institutions from their legal and professional responsibilities. You are free to withdraw from the study at any time, and /or refrain from answering any questions you prefer to omit, without prejudice or consequence.

The University of Manitoba or University of Calgary may look at your research records to see that the research is being done in a safe and proper way.

This research has been approved by the Fort Garry Research Ethics Board. If you have any concerns or complaints about this project, you may contact any of the above-named persons or the Human Ethics Coordinator at 204-474-7122 or humanethics@umanitoba.ca. A copy of this consent form has been given to you to keep for your records and reference.

## Abbreviations

AUDIT: Alcohol Use Disorder Identification Test
BEAM: Building Emotion Awareness and Mental Health
BFI-10: Big Five Inventory-10
CAD: Canadian dollars
COVID-19: Coronavirus disease 2019
CSI-4: 4-item Couple Satisfaction Index
DSI-SS: Depression Symptom Index Suicidality Subscale
ECBQ: Early Child Behavior Questionnaire
CUDIT-R: Cannabis Use Disorder Identification Test - Revised
GAD-7: Generalized Anxiety Disorder – 7 item
ICMJE: International Committee of Medical Journal Editors
ITT: Intent-to-treat
MAUQ: mHealth App Usability Questionnaire
ORBIT: Obesity-Related Behavioral Intervention Trials
PACER: Parental Assistance with Child Emotion Regulation
PARYC: Parenting Young Children Measure
PHIA: Personal Health Information Act
PHQ-9: Patient Health Questionnaire
PIs: Principal investigators
PPSC: Preschool Pediatric Symptom Checklist
PROMIS: Patient-Reported Outcomes Measurement Information System
PSI: Parenting Stress Index
RCT: Randomized controlled trial
REDCap: Research Electronic Data Capture
SCS-SF: Self-Compassion Scale Short Form
SISE: Single Item Self-Esteem Scale
SPIRIT: Standard Protocol Items: Recommendations for Interventional Trials
SSEQ: Social Support Effectiveness Questionnaire
TAU: Treatment as usual

## References

[1] Hawryluck L, Gold WL, Robinson S, Pogorski S, Galea S, Styra R (2004). SARS control and psychological effects of quarantine, Toronto, Canada. Emerg Infect Dis.

[2] Wang C, Pan R, Wan X, Tan Y, Xu L, Ho CS (2020). Immediate psychological responses and associated factors during the initial stage of the 2019 coronavirus disease (COVID-19) epidemic among the general population in China. Int J Environ Res Public Health.

[3] Suwalska J, Napierała M, Bogdański P, Łojko D, Wszołek K, Suchowiak S (2021). Perinatal mental health during COVID-19 pandemic: an integrative review and implications for clinical practice. J Clin Med.

[4] Iyengar U, Jaiprakash B, Haitsuka H, Kim S (2021). One year into the pandemic: a systematic review of perinatal mental health outcomes during COVID-19. Front Psychiatry.

[5] Connor J, Madhavan S, Mokashi M, Amanuel H, Johnson NR, Pace LE (2020). Health risks and outcomes that disproportionately affect women during the COVID-19 pandemic: a review. Soc Sci Med.

[6] Wade M, Prime H, Johnson D, May SS, Jenkins JM, Browne DT (2021). The disparate impact of COVID-19 on the mental health of female and male caregivers. Soc Sci Med.

[7] Civic D, Holt VL (2000). Maternal depressive symptoms and child behavior problems in a nationally representative normal birthweight sample. Matern Child Health J.

[8] Horwitz SM, Briggs-Gowan MJ, Storfer-Isser A, Carter AS (2009). Persistence of maternal depressive symptoms throughout the early years of childhood. J Women's Health.

[9] Matthey S, Barnett B, Ungerer J, Waters B (2000). Paternal and maternal depressed mood during the transition to parenthood. J Affect Disord.

[10] Wang L, Wu T, Anderson JL, Florence JE (2011). Prevalence and risk factors of maternal depression during the first three years of child rearing. J Women's Health.

[11] Stein A, Pearson RM, Goodman SH, Rapa E, Rahman A, McCallum M (2014). Effects of perinatal mental disorders on the fetus and child. Lancet..

[12] Zubaran C, Foresti K (2013). The correlation between breastfeeding self-efficacy and maternal postpartum depression in southern Brazil. Sex Reprod Healthc.

[13] Racine N, Hetherington E, McArthur BA, McDonald S, Edwards S, Tough S (2021). Maternal depressive and anxiety symptoms before and during the COVID-19 pandemic in Canada: a longitudinal analysis. Lancet Psychiatry.

[14] Hessami K, Romanelli C, Chiurazzi M, Cozzolino M. COVID-19 pandemic and maternal mental health: a systematic review and meta-analysis. J Matern Fetal Neonatal Med. 2020:1–8. 10.1080/14767058.2020.1843155 33135523

[15] Loret de Mola C, Martins-Silva T, Carpena MX, Del-Ponte B, Blumenberg C, Martins RC (2021). Maternal mental health before and during the COVID-19 pandemic in the 2019 Rio Grande birth cohort. Braz J Psychiatry.

[16] Lebel C, MacKinnon A, Bagshawe M, Tomfohr-Madsen L, Giesbrecht G (2020). Elevated depression and anxiety symptoms among pregnant individuals during the COVID-19 pandemic. J Affect Disord.

[17] Tomfohr-Madsen L, Giesbrecht G, Lebel C, Racine N, Madigan S (2021). Depression and anxiety in pregnancy during COVID-19: a rapid review and meta-analysis. Psychiatry Res.

[18] Lancaster CA, Gold KJ, Flynn HA, Yoo H, Marcus SM, Davis MM (2010). Risk factors for depressive symptoms during pregnancy: a systematic review. Am J Obstet Gynecol.

[19] Ghaedrahmati M, Kazemi A, Kheirabadi G, Ebrahimi A, Bahrami M (2017). Postpartum depression risk factors: a narrative review. J Educ Health Promot.

[20] Moussavi S, Chatterji S, Verdes E, Tandon A, Patel V, Ustun B (2007). Depression, chronic diseases, and decrements in health: results from the World Health Surveys. Lancet..

[21] Bauer A, Knapp M, Parsonage M (2016). Lifetime costs of perinatal anxiety and depression. J Affect Disord.

[22] Chivers BR, Garad RM, Boyle JA, Skouteris H, Teede HJ, Harrison CL (2020). Perinatal distress during COVID-19: thematic analysis of an online parenting forum. J Med Internet Res.

[23] Rasic D, Hajek T, Alda M, Uher R (2014). Risk of mental illness in offspring of parents with schizophrenia, bipolar disorder, and major depressive disorder: a meta-analysis of family high-risk studies. Schizophr Bull.

[24] Wachs TD, Black MM, Engle PL (2009). Maternal depression: a global threat to children’s health, development, and behavior and to human rights. Child Dev Perspect.

[25] Rahman A, Surkan PJ, Cayetano CE, Rwagatare P, Dickson KE (2013). Grand challenges: integrating maternal mental health into maternal and child health programmes. PLoS Med.

[26] Giesbrecht G, Lebel C, Dennis C, Tough SC, McDonald S, Tomfohr-Madsen L (2022). Increased risk for developmental delay among babies born during the pandemic.

[27] Bridgett DJ, Burt NM, Edwards ES, Deater-Deckard K (2015). Intergenerational transmission of self-regulation: a multidisciplinary review and integrative conceptual framework. Psychol Bull.

[28] Calkins SD, Dollar JM (2014). Emotion: Commentary: a biopsychosocial perspective on maternal psychopathology and the development of child emotion regulation. J Personal Disord.

[29] Thanhäuser M, Lemmer G, de Girolamo G, Christiansen H (2017). Do preventive interventions for children of mentally ill parents work? Results of a systematic review and meta-analysis. Curr Opin Psychiatry.

[30] Shonkoff JP, Fisher PA (2013). Rethinking evidence-based practice and two-generation programs to create the future of early childhood policy. Dev Psychopathol.

[31] Glasheen C, Richardson GA, Fabio A (2010). A systematic review of the effects of postnatal maternal anxiety on children. Arch Womens Mental Health.

[32] Rees S, Channon S, Waters CS (2019). The impact of maternal prenatal and postnatal anxiety on children’s emotional problems: a systematic review. Eur Child Adolesc Psychiatry.

[33] Schwartz H, McCusker J, Law S, Zelkowitz P, Somera J, Singh S (2021). Perinatal mental healthcare needs among women at a community hospital. J Obstet Gynaecol Can..

[34] Wind TR, Rijkeboer M, Andersson G, Riper H (2020). The COVID-19 pandemic: the ‘black swan’ for mental health care and a turning point for e-health. Internet Interv.

[35] Cameron EE, Joyce KM, Delaquis CP, Reynolds K, Protudjer JL, Roos LE (2020). Maternal psychological distress & mental health service use during the COVID-19 pandemic. J Affect Disord.

[36] Roos LE, Salisbury M, Penner-Goeke L, Cameron EE, Protudjer JL, Giuliano R (2021). Supporting families to protect child health: parenting quality and household needs during the COVID-19 pandemic. PLoS One.

[37] Viveiros CJ, Darling EK (2018). Barriers and facilitators of accessing perinatal mental health services: the perspectives of women receiving continuity of care midwifery. Midwifery..

[38] Shorey S, Chee CYI, Ng ED, Lau Y, Dennis C-L, Chan YH (2019). Evaluation of a technology-based peer-support intervention program for preventing postnatal depression (part 1): randomized controlled trial. J Med Internet Res.

[39] Clarke K, King M, Prost A (2013). Psychosocial interventions for perinatal common mental disorders delivered by providers who are not mental health specialists in low-and middle-income countries: a systematic review and meta-analysis. PLoS Med.

[40] Dennis C-L, Chung-Lee L (2006). Postpartum depression help-seeking barriers and maternal treatment preferences: a qualitative systematic review. Birth..

[41] Czajkowski SM, Powell LH, Adler N, Naar-King S, Reynolds KD, Hunter CM (2015). From ideas to efficacy: the ORBIT model for developing behavioral treatments for chronic diseases. Health Psychol.

[42] Walker LO, Czajkowski SM (2019). Designing interventions to improve the health of women using the ORBIT model. J Obstet Gynecol Neonatal Nurs.

[43] MacKinnon AL, Simpson KM, Salisbury MR, Penner-Goeke L, Rioux C, Giesbrecht G (2022). Building Emotional Awareness and Mental Health (BEAM): a pilot randomized controlled trial of an App-based program for mothers of toddlers.

[44] Harris PA, Taylor R, Minor BL, Elliott V, Fernandez M, O’Neal L (2019). The REDCap Consortium: building an international community of software platform partners. J Biomed Inform.

[45] Harris PA, Taylor R, Thielke R, Payne J, Gonzalez N, Conde JG (2009). Research electronic data capture (REDCap)—a metadata-driven methodology and workflow process for providing translational research informatics support. J Biomed Inform.

[46] Whiteford HA, Harris M, McKeon G, Baxter A, Pennell C, Barendregt J (2013). Estimating remission from untreated major depression: a systematic review and meta-analysis. Psychol Med.

[47] Barlow DH, Farchione TJ, Bullis JR, Gallagher MW, Murray-Latin H, Sauer-Zavala S (2017). The unified protocol for transdiagnostic treatment of emotional disorders compared with diagnosis-specific protocols for anxiety disorders: a randomized clinical trial. JAMA Psychiatry.

[48] Cassiello-Robbins C, Southward MW, Tirpak JW, Sauer-Zavala S (2020). A systematic review of Unified Protocol applications with adult populations: facilitating widespread dissemination via adaptability. Clin Psychol Rev.

[49] MacBeth A, Gumley A (2012). Exploring compassion: a meta-analysis of the association between self-compassion and psychopathology. Clin Psychol Rev.

[50] Ferrari M, Hunt C, Harrysunker A, Abbott MJ, Beath AP, Einstein DA (2019). Self-compassion interventions and psychosocial outcomes: a meta-analysis of RCTs. Mindfulness..

[51] Schueller SM, Neary M, Lai J, Epstein DA (2021). Understanding people’s use of and perspectives on mood-tracking apps: interview study. JMIR Mental Health.

[52] Psychologists CA (2018). Practice guideline - telepsychology services.

[53] Linardon J, Cuijpers P, Carlbring P, Messer M, Fuller-Tyszkiewicz M (2019). The efficacy of app-supported smartphone interventions for mental health problems: a meta-analysis of randomized controlled trials. World Psychiatry.

[54] Gan DZ, McGillivray L, Han J, Christensen H, Torok M (2021). Effect of engagement with digital interventions on mental health outcomes: a systematic review and meta-analysis. Front Digit Health.

[55] Thongseiratch T, Leijten P, Melendez-Torres GJ (2020). Online parent programs for children’s behavioral problems: a meta-analytic review. Eur Child Adolesc Psychiatry.

[56] Alkhaldi G, Hamilton FL, Lau R, Webster R, Michie S, Murray E (2016). The effectiveness of prompts to promote engagement with digital interventions: a systematic review. J Med Internet Res.

[57] Kroenke K, Spitzer RL, Williams JB (2001). The PHQ-9: validity of a brief depression severity measure. J Gen Intern Med.

[58] Sidebottom AC, Harrison PA, Godecker A, Kim H (2012). Validation of the Patient Health Questionnaire (PHQ)-9 for prenatal depression screening. Arch Womens Mental Health.

[59] Spitzer RL, Kroenke K, Williams JB, Löwe B (2006). A brief measure for assessing generalized anxiety disorder: the GAD-7. Arch Intern Med.

[60] Abidin RR, Abidin RR (1990). Parenting Stress Index (PSI).

[61] Cohodes EM, Preece DA, McCauley S, Rogers MK, Gross JJ, Gee DG (2022). Development and validation of the Parental Assistance with Child Emotion Regulation (PACER) Questionnaire. Res Child Adolesc Psychopathol..

[62] Arnold DS, O’leary SG, Wolff LS, Acker MM (1993). The Parenting Scale: a measure of dysfunctional parenting in discipline situations. Psychol Assess.

[63] McEachern AD, Dishion TJ, Weaver CM, Shaw DS, Wilson MN, Gardner F (2012). Parenting Young Children (PARYC): validation of a self-report parenting measure. J Child Fam Stud.

[64] Rini C, Schetter CD, Hobel CJ, Glynn LM, Sandman CA (2006). Effective social support: antecedents and consequences of partner support during pregnancy. Pers Relationsh.

[65] Funk JL, Rogge RD. Testing the ruler with item response theory: increasing presocial support and communitycision of measurement for relationship satisfaction with the Couples Satisfaction Index. J Fam Psychol. 2007;21(4):572. 10.1037/0893-3200.21.4.572 18179329

[66] Cella D, Riley W, Stone A, Rothrock N, Reeve B, Yount S (2010). The Patient-Reported Outcomes Measurement Information System (PROMIS) developed and tested its first wave of adult self-reported health outcome item banks: 2005–2008. J Clin Epidemiol.

[67] Sutton E, Schonert-Reichl KA, Wu AD, Lawlor MS (2018). Evaluating the reliability and validity of the self-compassion scale short form adapted for children ages 8–12. Child Indic Res.

[68] Robins RW, Hendin HM, Trzesniewski KH (2001). Measuring global self-esteem: construct validation of a single-item measure and the Rosenberg Self-Esteem Scale. Personal Soc Psychol Bull.

[69] Hanish AE, Lin-Dyken DC, Han JC (2017). PROMIS sleep disturbance and sleep-related impairment in adolescents: examining psychometrics using self-report and actigraphy. Nurs Res.

[70] Joiner T, Pfaff JJ, Acres JG (2002). A brief screening tool for suicidal symptoms in adolescents and young adults in general health settings: reliability and validity data from the Australian National General Practice Youth Suicide Prevention Project. Behav Res Ther.

[71] Saunders JB, Aasland OG, Babor TF, De La Fuente JR, Grant M (1993). Development of the alcohol use disorders identification test (AUDIT): WHO collaborative project on early detection of persons with harmful alcohol consumption-II. Addiction..

[72] Adamson SJ, Kay-Lambkin FJ, Baker AL, Lewin TJ, Thornton L, Kelly BJ (2010). An improved brief measure of cannabis misuse: the Cannabis Use Disorders Identification Test-Revised (CUDIT-R). Drug Alcohol Depend.

[73] Sheldrick RC, Henson BS, Merchant S, Neger EN, Murphy JM, Perrin EC (2012). The Preschool Pediatric Symptom Checklist (PPSC): development and initial validation of a new social/emotional screening instrument. Acad Pediatr.

[74] Putnam SP, Rothbart MK (2006). Development of short and very short forms of the Children’s Behavior Questionnaire. J Pers Assess.

[75] Fitabase (2022). What is Fitabase?.

[76] Zhou L, Bao J, Setiawan IMA, Saptono A, Parmanto B (2019). The mHealth APP Usability Questionnaire (MAUQ): development and validation study. JMIR Mhealth Uhealth.

[77] Rammstedt B, John OP (2007). Measuring personality in one minute or less: a 10 item short version of the Big Five Inventory in English and German. J Res Pers.

[78] Moshe I, Terhorst Y, Philippi P, Domhardt M, Cuijpers P, Cristea I (2021). Digital interventions for the treatment of depression: a meta-analytic review. Psychol Bull.

[79] Faul F, Erdfelder E, Lang A-G, Buchner A (2007). G* Power 3: a flexible statistical power analysis program for the social, behavioral, and biomedical sciences. Behav Res Methods.

[80] Hedges LV (2007). Effect sizes in cluster-randomized designs.

[81] Dunlap WP, Cortina JM, Vaslow JB, Burke MJ (1996). Meta-analysis of experiments with matched groups or repeated measures designs. Psychol Methods.

[82] Patton MQ (2002). Qualitative research and evaluation methods.

[83] Schulz KF, Altman DG, Moher D (2010). CONSORT 2010 statement: updated guidelines for reporting parallel group randomized trials. Ann Intern Med.

[84] Kwok O-M, Underhill AT, Berry JW, Luo W, Elliott TR, Yoon M (2008). Analyzing longitudinal data with multilevel models: an example with individuals living with lower extremity intra-articular fractures. Rehabil Psychol.

[85] Enders CK (2010). Applied missing data analysis.

