# Correction: Building Emotional Awareness and Mental Health (BEAM): study protocol for a phase III randomized controlled trial of the BEAM app-based program for mothers of children 18–36 months

**DOI:** 10.1186/s13063-022-06760-5

**Published:** 2022-09-30

**Authors:** E. Bailin Xie, Kaeley M. Simpson, Kristin A. Reynolds, Ryan J. Giuliano, Jennifer L. P. Protudjer, Melanie Soderstrom, Shannon Sauer-Zavala, Gerald F. Giesbrecht, Catherine Lebel, Anna L. Mackinnon, Charlie Rioux, Lara Penner-Goeke, Makayla Freeman, Marlee R. Salisbury, Lianne Tomfohr-Madsen, Leslie E. Roos

**Affiliations:** 1 Department of Psychology, University of Calgary, 2500 University Dr. NW, Calgary, AB T2N 1N4 Canada; 2 University of Manitoba, Winnipeg, Canada; 3 Children’s Hospital Research Institute of Manitoba, Winnipeg, Canada; 4 University of Kentucky, Lexington, USA; 5 York University, Toronto, Canada

**Correction: Trials 23, 741 (2022)**

**https://doi.org/10.1186/s13063-022-06512-5**

Following the publication of the original article [1], we were notified that due to a technical error, Appendix 1 was covering the Discussion part of the article.

The original article has been corrected.
